# Importance of mixotrophic flagellates during the ice-free season in lakes located along an elevational gradient

**DOI:** 10.1007/s00027-019-0643-2

**Published:** 2019-04-16

**Authors:** Anna Waibel, Hannes Peter, Ruben Sommaruga

**Affiliations:** 1 Department of Ecology, Lake and Glacier Research Group, University of Innsbruck, Technikerstr. 25, 6020 Innsbruck, Austria; 2 Present Address: Stream Biofilm and Ecosystem Research Laboratory, Ecole Polytechnique Federale de Lausanne, 1015 Lausanne, Switzerland

**Keywords:** Mixotrophs, Phagotrophy, Phytoflagellates, Food webs, Mountain lakes

## Abstract

Mixotrophy seems to be widespread among phytoplankton, but whether this strategy is more relevant in oligotrophic lakes remains unclear. Here, we tested the hypothesis that the relative abundance of mixotrophic flagellates in lakes increases along an elevational gradient paralleling increasingly oligotrophic conditions. For this purpose, 12 lakes located between 575 and 2796 m above sea level were sampled in summer and fall to include two different seasonal windows in phytoplankton dynamics and environmental conditions. The degree of mixotrophy in phytoplankton was estimated in tracer experiments using fluorescently-labeled bacteria and done with composite samples collected in the euphotic zone and in samples obtained from the chlorophyll-*a* maximum. The results indicated the existence of a positive trend particularly in summer in the relative abundance of mixotrophic flagellates with elevation, however, this trend was not linear, and exceptions along the elevational gradient were found. Changes in the relative abundance of mixotrophic flagellates were related with significant changes in water transparency, DOC and phosphorus concentrations, as well as in bacterial and flagellate abundance. Overall, our results reveal that the harsh growth conditions found in oligotrophic high mountain lakes favor a mixotrophic trophic strategy among phytoplankton.

**Electronic supplementary material:**

The online version of this article (10.1007/s00027-019-0643-2) contains supplementary material, which is available to authorized users.

## Introduction

Many microbial planktonic organisms combine photoautotrophic and heterotrophic (e.g., phagotrophic) nutritional modes and are referred as mixotrophs. Although the existence of mixotrophic algal species was recognized long ago (Hofeneder 1930), the importance of this nutritional strategy in food webs was much later acknowledged (Boraas et al. 1988; Bird and Kalff 1989; Ward and Follows 2016). Most flagellated marine and freshwater phytoplankton groups are known to include mixotrophic representatives able to ingest different preys (Stoecker et al. 2009), for example within chrysophytes (Rothhaupt 1996a; Rottberger et al. 2013), dinophytes (Stoecker 1999), cryptophytes (Laybourn-Parry et al. 2005), and euglenophytes (Epstein and Shiaris 1992). Chrysophytes, however, are usually described as the dominant group of mixotrophic phytoplankton in oligotrophic lakes (Ptacnik et al. 2008; Saad et al. 2016), whereas mixotrophic cryptophytes seems to dominate in eutrophic lakes (Saad et al. 2016).

Mixotrophic flagellates can be at least equally important as heterotrophic and photoautotrophic ones in terms of abundance (Sanders et al. 2000), biomass (Bergström et al. 2003) and grazing rates (Domaizon et al. 2003), and though they are found in aquatic ecosystems of different trophic state, they seem to prevail in oligotrophic conditions (Domaizon et al. 2003; Anneville et al. 2005; Hartmann et al. 2012; Fisher et al. 2017). However, other studies have found that at least under the ice cover, mixotrophic phytoplankton were more important in a mesotrophic lake rather than in oligotrophic or highly eutrophic ones (Berninger et al. 1992).

The causes why mixotrophy in phytoplankton offers a competitive advantage over strict photoautotrophy, particularly in oligotrophic environments, seem to be multiple and are not easy to disentangle (e.g., Wilken et al. 2018). Certainly, the ability of constitutive mixotrophs (i.e., those synthesizing their own chloroplasts, Mitra et al. 2014) to access multiple and substitutional resources from additional carbon and nutrient sources, for example, by grazing on bacteria is crucial to attain population growth under limiting conditions. Further, phagotrophy can provide essential growth factors and trace nutrients to phytoplankton that otherwise are limiting in the dissolved fraction (Caron et al. 1993; Maranger et al. 1998). However, maintaining two different metabolic systems may also imply higher metabolic costs (Tittel et al. 2003; Raven et al. 2009; Ward et al. 2011). In fact, the balance between investments in photosynthesis and phagotrophy may change over time (Berge et al. 2016) and it seems to largely depend on light intensity and quality (Li et al. 2000; Pålsson and Graneli 2003; Wilken et al. 2018).

In marine oligotrophic surface waters, high-light conditions, but also low predatory losses seems to explain the success of mixotrophic flagellates compared to heterotrophic ones (Fisher et al. 2017). However, oligotrophic surface waters are not only characterized by high light levels, but also by high UV radiation (Laurion et al. 2000), which is an important stressor for many species (Sommaruga 2001). In this context, mixotrophy in lake phytoplankton has been proposed to be an adaptive strategy to compensate for negative UV effects on photosynthetic and nutrient uptake rates (Medina-Sanchéz et al. 2004).

Overall, although mixotrophic phytoplankton are acknowledged to be ubiquitous in freshwater and marine ecosystems (Stoecker 1998), the environmental conditions that favor their prevalence are not well understood (Berge et al. 2016). In this regard, lakes located along an elevational gradient are suitable study objects to test the importance of mixotrophy in phytoplankton because it entails several strong environmental gradients. For example, most lowland lakes have low water transparency due to their high concentration of (colored) dissolved organic carbon (DOC), whereas most mountain lakes, particularly those located above the treeline (i.e., alpine lakes), show high transparency and light availability coupled to their low DOC concentration (Rose et al. 2009). In addition, the concentration of limiting nutrients such as phosphorus is also higher in lowland lakes than in mountain ones (Stenzel et al. 2017). Though phagotrophy by phytoflagellates is acknowledged to be an important process in high mountain lakes (Callieri et al. 2006), it is has never been tested whether mixotrophic flagellates become more relevant among phytoplankton along the elevational gradient.

In this study, our goal was to test whether the proportion of mixotrophic flagellates increases along the elevational gradient that parallel an increase in oligotrophy. We sampled 12 lakes during two different periods along an elevational gradient to reveal the relative importance of mixotrophic flagellates collected from the chlorophyll-*a* maximum and from composite samples of the whole water column.

## Materials and methods

### Sampling and physico-chemical and chlorophyll-a measurements

The 12 lakes were located between 575 and 2796 m above sea level in Tyrol, Austria (Table S1, see also Stenzel et al. (2017) for other lake characteristics such as lake area and maximum depth). The lakes were sampled twice during the ice-free season. The first sampling was done in July during the summer stratification period and when a deep chlorophyll-*a* (chl-*a*) maximum typically develops, particularly in alpine lakes. The second sampling was done in October during the fall overturn. Sampling in two alpine lakes (Mutterbergersee and Schwarzsee ob Sölden) was only possible in October because a helicopter is needed to reach these remote ecosystems. Sampling was done in all cases over the deepest point of the lakes from a boat with a 5 L Schindler-Patalas sampler.

A multiparameter probe (YSI 6600 V2) was used to make profiles in the water column for temperature, conductivity, pH, oxygen, and chl-*a* fluorescence. The latter measurement was used to define the depth of the chl-*a* maximum. The intensity of the photosynthetically available radiation (PAR) was measured at 0.5 m depth intervals with a photometer (LI-14000, LI-COR). Water samples were collected at the chl-*a* maximum and additionally a composite water sample was collected to obtain a representative picture for the whole water column. For this purpose, the same volume of lake water collected at 1 m intervals was pooled. To homogenize variability caused by different light penetration among lakes, water for the composite sample was collected until the 1% attenuation depth of PAR was reached. In October, a chl-*a* maximum was not detected in three of the lakes (i.e., Baggersee, Obernbergersee, and Oberer Plenderlessee).

Concentrations of dissolved organic carbon (DOC), dissolved nitrogen (DN), ammonium (NH_4_), nitrate (NO_3_), total phosphorus (TP), total dissolved phosphorus (TDP), and chlorophyll-a (chl-a) were measured for both types of samples. The dissolved fraction was defined as the filtrate passing through a glass fiber filter (GF/F Whatman), pre-combusted at 450 °C for 2 h. DOC was measured in a total carbon analyzer (Shimadzu TOC—V_CPH_), DN was measured in a total nitrogen measuring unit (Shimadzu TNM–1), NH_4_ and NO_3_ were measured by ion chromatography (Dionex ICS–1000/1100), TP (unfiltered samples) and TDP were measured after the blue molybdate method (Vogler 1966), and chl-a was extracted with alkaline acetone and measured after Lorenzen (1967).

### Experiments to assess the proportion of mixotrophic flagellates

Fluorescently-labeled bacteria (FLB) were used as a prey tracer to detect mixotrophic flagellates under defined experimental conditions. Mixotrophic flagellates potentially feeding on other algae were not assessed. In order to prepare the FLB, natural bacteria were obtained from a *Spumella* sp. culture to assure edibility and comparability among experiments in the different lakes and stained with DTAF (-5-([4,6-dichlorotriazin-2-yl]amino) fluorescein) according to Sherr et al. (1987). Three replicate subsamples of 100 ml were taken in each lake from both the chl-a maximum and the composite sample. The water was first screened through a plankton net of 100 μm mesh size to remove large zooplankton and then, FLB were added to the samples to represent ~ 30% of the natural bacterial abundance. Bottles were incubated at the in situ water temperature (chl-*a* maximum) or average water temperature for the water column (composite sample). In July, the samples were incubated for 2 h in the field under ca. 50% PAR, using a double neutral density net to avoid photoinhibition. Controls fixed with formalin were included to check for the equivocal determination of FLB inside flagellates, but they were in all cases negative. After incubation, the samples were fixed according to Sherr et al. (1987) with a final concentration of 0.5% alkaline lugol solution and 3% borate-buffered formalin. In October and because of the low water temperatures, particularly in the high elevation lakes, the incubation lasted for 12 h and therefore, the experiments were done in a climate chamber equipped with light provided by a luminescent set of 4 lamps (PAR: 80 μmol m^−2^ s^−1^). This incubation time was based on results from an experiment done in one of the lakes (Gossenköllesee) where different incubation times (2, 4, 8, 12, and 24 h) were tested. Although this incubation time might not be the optimal in all other lakes, we argue that it make the results more comparable than using different times for each lake. Nevertheless, the results of the experiments from July and October are not directly comparable and thus, differences were not tested.

### Quantification of bacteria, phytoplankton, and flagellates with and without ingested bacteria

The samples from the FLB experiments were filtered onto 1 μm pore size black polycarbonate filters and stained with DAPI (4′6-diamidino-2-phenylindole). Under the epifluorescence microscope (Axiophot 2, Zeiss), 100 flagellates were inspected per filter, and the number of apochlorotic (i.e., heterotrophic) and chloroplast-containing (i.e., photoautotrophic) flagellates and their respective proportion with ingested FLB was determined using different Ex/Em filter sets as described in Sherr et al. (1993). Briefly, a flagellate cell was identified using the DAPI signal, then the cell was inspected for the presence of chl-a autofluorescence and finally for ingested FLB. Those flagellates containing chloroplasts and ingested FLB were quantified as mixotrophic ones. The abundance of bacteria and phytoplankton was counted by flow cytometry (MoFlo Astrios High Speed Cell Sorter, Beckman & Coulter). The abundance of FLB was counted first and then the sample was stained with SYTO 13 and counted again, to obtain the abundance of bacteria by difference. As a reference, a known number of 1.0 μm carboxylated yellow-green fluorescent beads (505/515 Ex/Em, FluoSpheres, Invitrogen) was added to the samples. Bacteria were identified in scatterplots of log-transformed height signals of side scatter of the 488 nm laser versus fluorescence at 513/26. For phytoplankton counting, the sidescatter signal was plotted against the autofluorescence signal detected by the 561-692/275 detector. In this case, 1.0 μm carboxylated red fluorescent (580/605 Ex/Em) reference beads (FluoSpheres Invitrogen) were used. At least 500–1000 phytoplankton cells per sample were counted. The abundance of reference beads was determined by epifluorescence microscopy.

### Statistical analysis

Statistically significance differences between July and October for environmental parameters and for bacterial and phytoplankton abundance were assessed using paired *t* tests and the free software PAST (Hammer et al. 2001). Normal distribution of observations and homogeneity of variance were previously checked. Homogeneity of variance was not given for all comparisons and in this case, we used the unequal-variance Welch-test. In the statistical analyses, the term “Temperature” refers to the mean of water temperatures from all sampled depths for the composite data and the temperature at the depth of the chl-*a* maximum. The term PAR refers either to the 1% attenuation depth for PAR for the composite data or the percentage of surface PAR at the depth of the chl-*a* maximum. We used locally estimated scatterplot smoothing (LOESS) implemented in R to visualize non-linear trends along the elevational gradient in the dataset. Since many environmental parameters change in a collinear way along the elevational gradient (Supporting Figure S1), we used partial least square regression (PLSR) to assess the relative importance of environmental factors in explaining the variation of relative abundance of mixotrophic flagellates. For PLSR, the data were log-transformed and models were evaluated using seven leave-one-out segments. For July, the model considered five components, for October the model considered eight components. PLSR and the Correlation Plots (CP) were prepared using the package pls in R (R Core Team 2017).

## Results

**Fig. 1:**
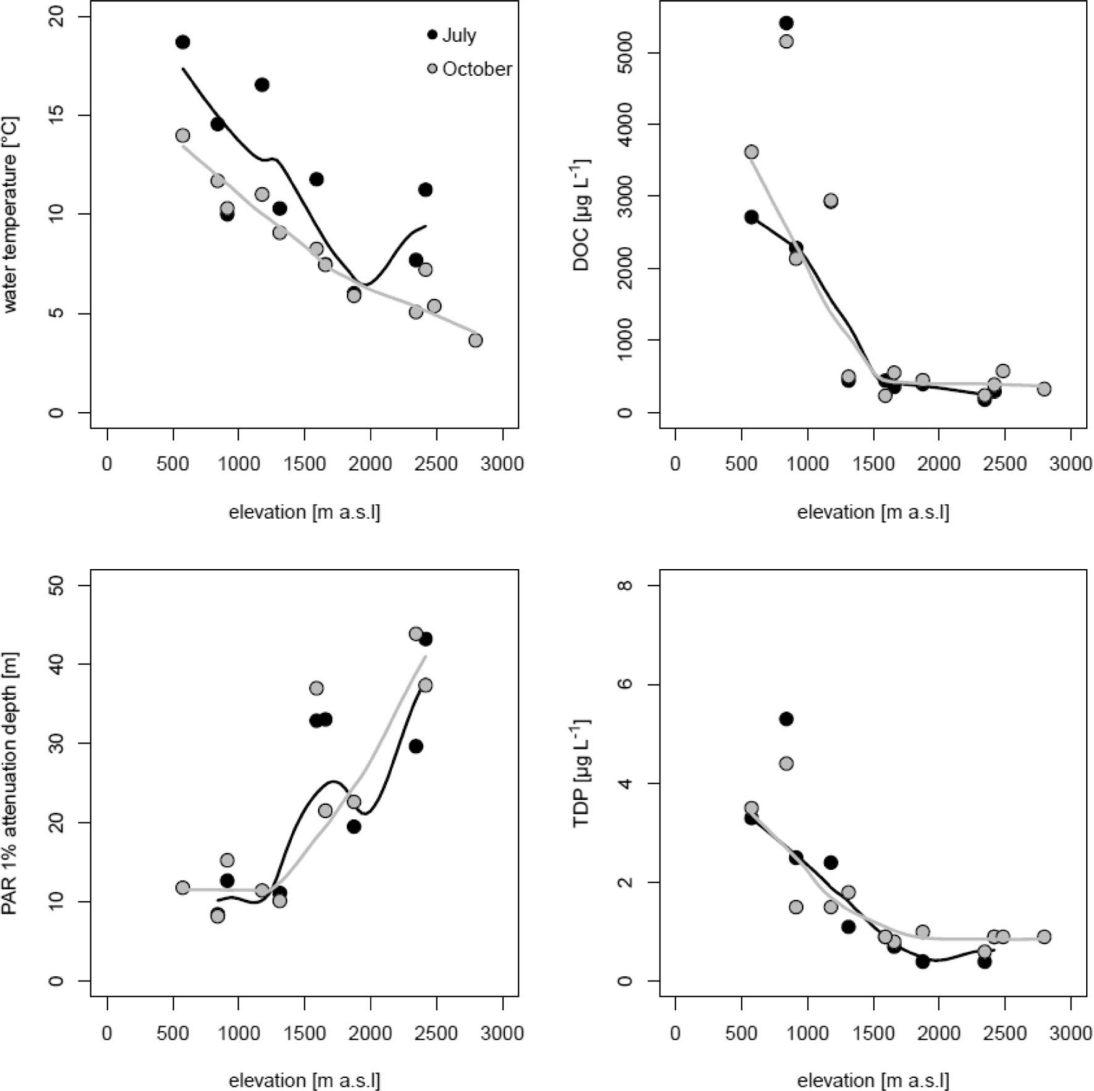
Key environmental parameters along the elevational gradient in July and October. Shown are measurements from composite water samples. The lines represent locally estimated scatterplot smoothing (loess) fits to the data data

Most environmental parameters showed marked changes along the elevational gradient in July and October (Fig. 1) and were highly correlated with elevation (Supporting Fig. S1). For instance, DOC concentrations ranged between 170 μg l^−1^ in the alpine Oberer Plenderlessee in July and 5400 μg L^−1^, in the lowland Lansersee in October (Fig. 1). Similarly, TDP concentrations ranged between 0.3 and 5.3 μg L^−1^ and the concentration of dissolved nitrogen was highest in the lakes below 1000 m a.s.l. (i.e., Lansersee: 4330 μg L^−1^ in July; Baggersee: 915 μg L^−1^ in July and 640 μg L^−1^ in October), whereas all other lakes had concentrations < 400 μg L^−1^. The concentration of TDP and DOC decreased with elevation (Fig. 1), whereas dissolved nitrogen showed little difference along the elevational gradient (Supporting Figs. S1 and S2). There was a marked shift in DOC concentration and the 1% PAR attenuation depth at ca. 1500 m a.s.l. (Figure 1). Below this elevation, DOC concentration tended to increase linearly, whereas the 1% PAR attenuation depth did not vary significantly. Above 1500 m a.s.l., DOC concentration remained invariant, whereas the 1% PAR attenuation depth increased continuously with elevation. This shift was even more pronounced in October.

Water temperature in July was significantly different (paired *t* test) from those in October, whereas the other parameters were not (Table S2). In July, the water column was stratified, whereas in October, the lakes were already mixed. The 1% attenuation depth for PAR ranged between 8 and 15 m in lakes at low elevation, but between 10 and 43 m in the mountain ones (Fig. 1). The attenuation depth was negatively and significantly correlated with the DOC concentration (Pearson correlation, *r* = − 0.67, *p* < 0.01, *n* = 33, SI Fig. 1). The chl-*a* concentration was significantly correlated with phytoplankton abundance (Pearson correlation, *r* = 0.330, *p* < 0.05, *n* = 37) and did not exhibit pronounced shifts along the elevational gradient (Supporting Figure S2).

### Bacterial and phytoplankton abundance

**Fig. 2:**
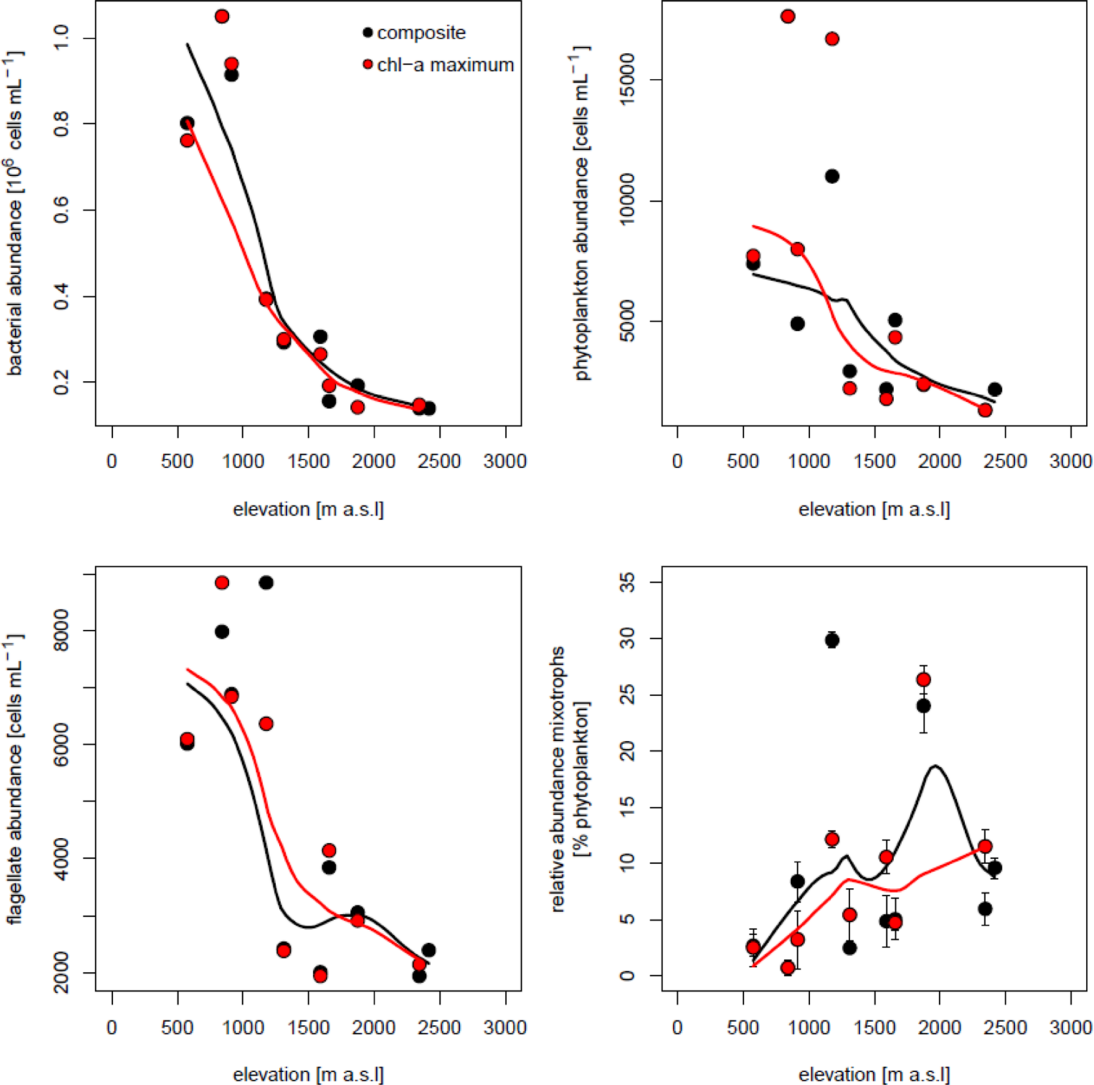
Changes in abundance of bacteria, phytoplankton and flagellates and in the relative abundance of mixotrophic flagellates (mixotrophs as percentage of phytoplankton abundance) along the elevational gradient in July. Shown are data for the composite water sample and for the depth of maximum chlorophyll-a concentration. The lines represent locally estimated scatterplot smoothing (loess) fits to the data. Error bars represent ± 1SD for the three parallels in the food tracer experiments

**Fig. 3:**
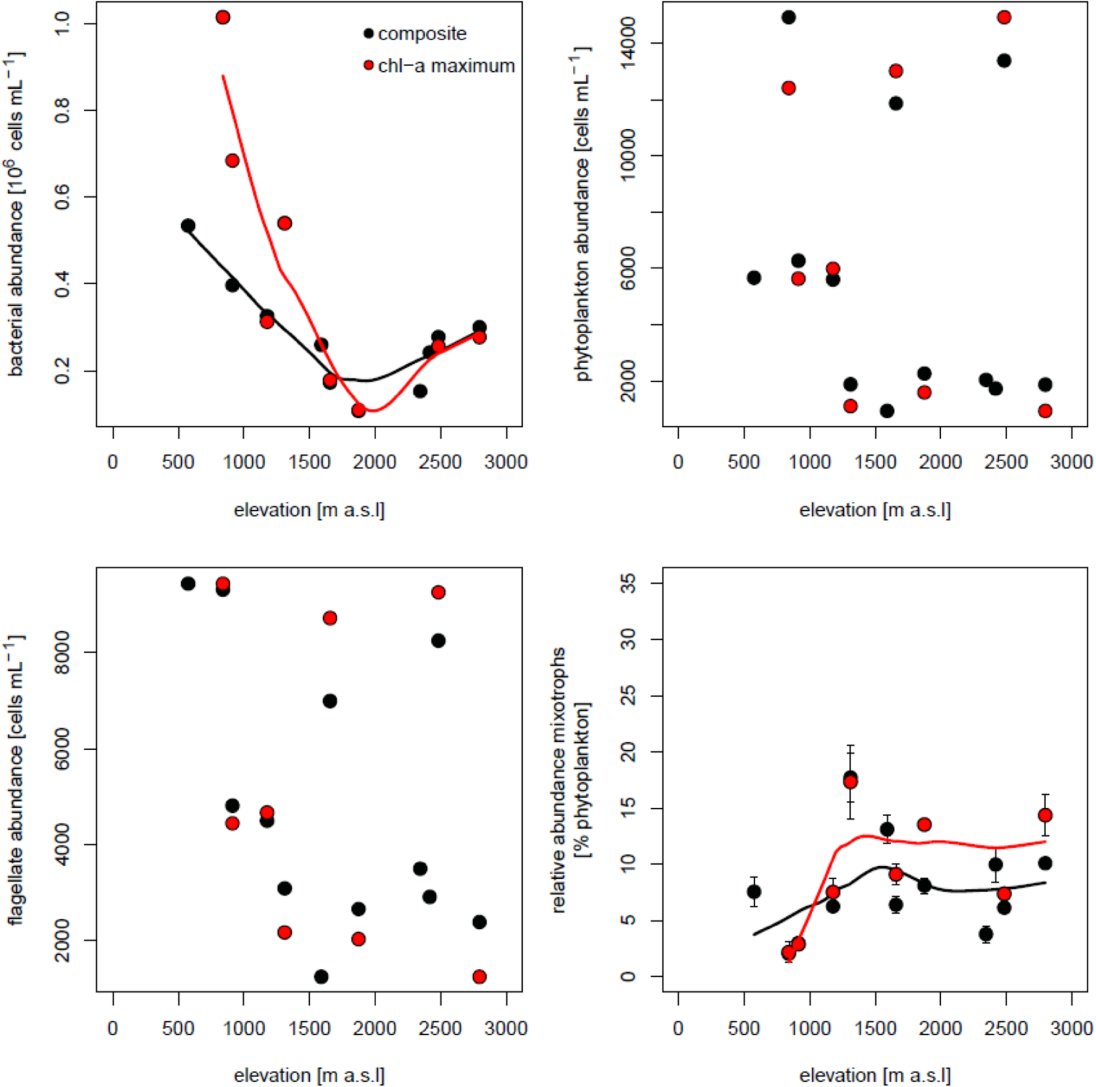
Changes in abundance of bacteria, phytoplankton and flagellates and in the relative abundance of mixotrophic flagellates (mixotrophs as percentage of phytoplankton abundance) along the elevational gradient in October. The lines represent locally estimated scatterplot smoothing (loess) fits to the data. Error bars represent ± 1SD for the three parallels in the food tracer experiments

Considering all data from the composite and chl-*a* maximum samples, bacterial abundance in lakes located below 1500 m a.s.l. ranged between 2.86 × 10^5^ and 1.05 × 10^6^ cells ml^−1^ in July and between 3.19 × 10^5^ and 1.01 × 10^6^ cells ml^−1^ in October, whereas in lakes above this elevation, values were < 4.00 × 10^5^ in July and < 5.45 × 10^5^ cells ml^−1^ in October (Figs. 2 and 3). Bacterial abundance was significantly correlated with the concentrations of DOC, TDP, DN, and with water temperature (Pearson correlation, *r* = 0.83, 0.93, 0.74, and 0.55, respectively, *p* < 0.01, *n* = 41). Phytoplankton abundance (Figs. 2 and 3) was usually higher in the low-lying lakes (between 4880 and 17,690 cells ml^−1^) than in the mountain ones (between 935 and 16,780 cells mL^−1^). However, in October, the high mountain lakes Drachensee and Mutterbergersee reached high phytoplankton abundances of > 11,000 cells mL^−1^ and the trend observed in July was absent (Fig. 3).

### Flagellates abundance

The total abundance of flagellates (i.e., sum of hetero- and phototrophic flagellates) decreased with elevation in July (Fig. 2), but this pattern was not clear in October (Fig. 3). Heterotrophic flagellates were significantly more abundant in lakes below 1500 m a.s.l. in July (*t* test, *t* = 3.44, *p* = 0.01) and in October (*t* test, *t* = 3.16, *p* = 0.01) than in lakes above this elevation (Supporting Fig. S3). The abundance of phytoflagellates estimated by epifluorescence microscopy was strongly correlated with that of total phytoplankton estimated using flow cytometry (*r* = 0.834, *p* < 0.01, *n* = 19 in July and *r* = 0.864, *p* < 0.01, *n* = 21 in October). On average for both months, the abundance of heterotrophic flagellates without ingested FLB (H–) was < 14%, an indication that the concentration of FLB and the incubation period used seemed appropriate.

### Relative abundance of mixotrophic flagellates

**Fig. 4:**
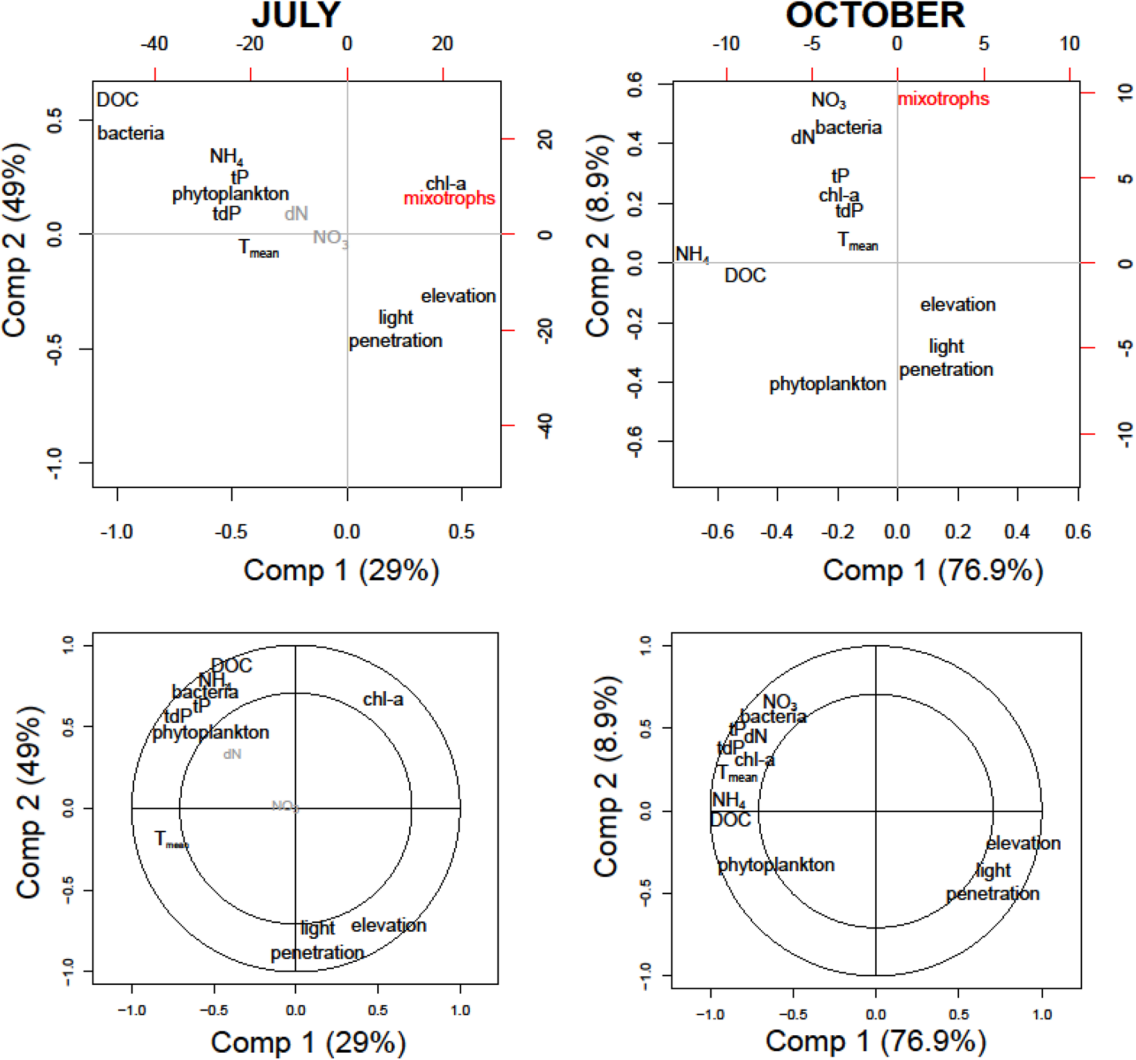
Partial least square regression (PLSR) showing the associations of relative abundance of mixotrophic phytoplankton with environmental parameter during the two sampling occasions (upper panels). Environmental factors close to the response variable (relative abundance of mixotrophic flagellates) indicates strong positive associations. In July, chl-a, elevation and light penetration were most strongly and positively associated with the relative abundance of mixotrophic flagellates. In October, the relative abundance of mixotrophic flagellates was rather associated with the trophic state. The correlation plot (lower panel) show the significance of the variables for the component axes of the PLS (lower panels). Grey font indicates non-significant factors

The relative abundance of mixotrophic flagellates in July and October increased with elevation in both the composite and deep chl-a maximum samples though with different patterns (Figs. 2 and 3). However, mixotrophic flagellates reached their maximum relative abundance (29.8%) in a lake located at 1180 m a.s.l. (Wildsee Seefeld). In July, the increase with elevation was more pronounced (particularly up to 2000 m a.s.l.) than in October when it flattened in lakes located above 1500 m a.s.l. Excluding the exceptionally high relative abundance of mixotrophic flagellates in Wildsee Seefeld, the difference in relative abundance between lakes located above and below 1500 m a.s.l. was statistically significant in July (*t* test, *t* = − 2.51, *p* = 0.04), but not in October (*t* test, *t* = − 0.52, *p* = 0.61). There was no significant difference between the relative abundance of mixotrophic flagellates in the composite water sample as compared to samples from the chl-*a* maximum (paired *t* test, *t* = − 0.44 *p* = 0.67 in July and *t* = − 0.60, *p* = 0.57 in October). These relationships were corroborated by the PLSR analysis (Fig. 4), which indicated that the variation in relative abundance of mixotrophic flagellates among lakes was best explained by a combination of elevation, light penetration (= PAR 1% attenuation depth), and chl-*a* (only in July). The correlation plots (Fig. 4) visualizes the significance of the variables for the component axes of the PLSR. All variables were significant, with the exception of dissolved nitrogen in July.

We also estimated the importance of mixotrophic flagellates considering only the abundance of autotrophic flagellates as a reference (Supporting Fig. S4). These results showed a similar pattern as when considering the total phytoplankton abundance though the relative abundance differed. For example in July, the maximum percentage of mixotrophic flagellates reached up to 46.3%, again in the composite sample of Wildsee Seefeld.

## Discussion

Our results indicate that the relative abundance of mixotrophic flagellates tended to increase along the elevational gradient (Figs. 2 and 3). Although the trends were complex and non-linear, they are remarkable considering that we used the same experimental conditions (i.e., time, light, prey type) to estimate the relative abundance of mixotrophic flagellates for all lakes. Further, the shift towards higher relative abundance of mixotrophic flagellates at higher elevations is even more surprising considering that mixotrophic taxa differ in their relative dependence on light and prey type (Gast et al. 2014).

The complexity of the trends observed may reflect non-linear changes in environmental conditions, such as those occurring around the treeline. Further, some lake specific characteristics may affect the trends observed as it seems to be the case of Wildsee Seefeld, the only lake in this series with an extensive littoral zone with emergent macrophytes, though it is difficult to pinpoint how this affects the degree of mixotrophy in phytoplankton.

In general, the relative importance of mixotrophic flagellates among phytoplankton was related with the trophic gradient of the studied lakes. This is supported by the significant negative relationship between the relative abundance of mixotrophic flagellates and the concentrations of total phosphorus and nitrogen (Fig. 4). In fact, the lowland lakes had higher concentrations of dissolved phosphorus and nitrogen than the high mountain lakes (Fig. 1 and Supporting Fig. S2). The mixotrophic strategy is successful when nutrients are limiting (Rothhaupt 1996b; Ward et al. 2011). In general, mixotrophic phytoplankton seem to be important in oligotrophic environments (Anneville et al. 2005; Domaizon et al. 2003, Hartmann et al. 2012). When nutrients are limiting, grazing on bacteria provides an important nutritional source, particularly of phosphorus (Bergström et al. 2003; Mitra et al. 2014). Due to the oligotrophic condition of most mountain lakes and the high atmospheric deposition of reactive nitrogen over the Alps, the N:P ratio in the dissolved fraction is extremely unbalanced (Pérez et al. 2006; Stenzel et al. 2017). However, as shown by a parallel study to this (Stenzel et al. 2017), the microbial fraction (largely dominated by bacteria) is more P-depleted (i.e., higher C:P) in lowland lakes than in those located above the treeline. Thus, mixotrophic flagellates in mountain lakes actually feed on bacteria with relative high phosphorus cell content that in turn may favor their growth. Further, mixotrophic flagellates only need to consume a fraction of the bacterial pool to complement their phosphorus demand (Ward et al. 2011).

The relative abundance of mixotrophic flagellates was also significantly influenced by the DOC concentration (Fig. 4). This is a central parameter in the context of mixotrophy because it affects bacterial production and, at the same time, light conditions (Wilken et al. 2018). However, the negative association of DOC and bacterial abundance with the relative proportion of mixotrophic flagellates (Fig. 4) suggests that prey availability was not the primary factor determining the importance of mixotrophy in the studied lakes. By contrast, light is probably more important because it directly affects the competitive interaction with heterotrophic flagellates (Mitra et al. 2014; Fisher et al. 2017). Nevertheless, mixotrophic species differ in their dependence on light and some taxa are known to grow in the dark when enough food is available, as well as many taxa, can grow in light without feeding (Jones 2000). Obviously, information on taxonomic identity of mixotrophic taxa and physiological experiments with isolates (Gast et al. 2018), as well as combination of different approaches (see for a review Beisner et al. 2019) are needed to understand what environmental factors are relevant in the field.

Considering that DOC concentration and PAR attenuation did not exhibit a simple linear relationship with elevation, but instead showed a shift in their trend around 1500 m a.s.l., we attribute these shifts to changes in catchment properties such as the percentage of vegetation cover that influences the optical characteristics of DOM (Laurion et al. 2000). In general, high mountain lakes had higher water transparency (Fig. 1) than lowland lakes as previously found (Laurion et al. 2000; Rose et al. 2009). Terrestrial vegetation above the treeline in temperate regions is scarce and consequently, the input of allochthonous organic matter declines and, as a result, the concentration of in-lake DOC (Sommaruga 2001). Therefore, light in this type of lakes is not limiting during the ice-free season, rather high PAR and UV levels can cause strong photoinhibition (Callieri et al. 2001). In this sense, phagotrophy in phytoplankton could be an important strategy to compensate for reduced photosynthetic rates under UV stress (Medina-Sanchéz et al. 2004).

Mountain lakes, particularly those located above the treeline, are typically high light/low nutrients environments. The light:nutrient hypothesis (Sterner et al. 1997) states that in environments with low nutrient concentrations and high light intensities, the stoichiometric composition of phytoplankton is not optimal for their consumers because of their high C:P ratio and low nutritional quality. However, mixotrophic phytoplankton seem to have superior food quality for herbivore zooplankton because their C:P ratio is lower and less variable than that of strictly autotrophic phytoplankton (Katechakis et al. 2005; Moorthi et al. 2017). The low C:P in mixotrophic phytoplankton is thought to be related to the extra supplement of phosphorus obtained through feeding on bacteria (Katechakis et al. 2005). Thus, in environments with low nutrient and high light conditions, mixotrophic flagellates could act as an important link to higher trophic levels (Ptacnik et al. 2004).

In lowland and less transparent lakes, the chl-*a* maximum was located near to the surface, whereas in the clear mountain lakes it was deeper and situated close to the lake’s bottom. However, we did not observe a significantly higher percentage of mixotrophic flagelattes at the chl-*a* maximum than on average for the whole water column. Although the deep-chlorophyll maximum is a common feature observed in clear mountain lakes, the reasons for it are not always clear. One explanation is that phytoplankton migrates to deep water layers during daytime to avoid inhibitory UV radiation levels (Rodhe et al. 1966; Sommaruga 2001). Another cause could be that nutrient concentrations are higher near the sediment, due to degrading processes below the thermocline (Saros et al. 2005). Further, Tittel et al. (2003) hypothesized that the mixotrophic grazing strategy of phytoplankton (when feeding on other algae such as in the case of *Ochromonas* sp. strain 1B3) is responsible for the deep-chlorophyll maximum in many aquatic environments.

We conclude that the increasing importance of mixotrophic flagellates along the elevational gradient derives from a combination of physicochemical factors and that they can reach higher relative abundances in most mountain lakes than in those located at the valley. Whereas mixotrophy is arguably an important, but relatively understudied ecological strategy, we identify clear and oligotrophic lakes at high elevation as potential hotspots for a mixotrophic trophic strategy of phytoplankton. The importance of mixotrophic flagellates in these lakes may even increase under the ice-cover (Sommaruga unpubl.) although results from previous studies are not conclusive (Berninger et al. 1992). Finally, considering that changes in both autochthonous or allochthonous DOC concentration (Parker et al. 2008, Wilken et al. 2018) favor the dominance of mixotrophic phytoplankton, climate change will probably alter the balance of phototrophic, mixotrophic and heterotrophic processes.

## Electronic supplementary material

Below is the link to the electronic supplementary material.

Supplementary material 1 (DOCX 635 kb)
