# Supplementary material for: Importance of mixotrophic flagellates during the ice-free season in lakes located along an elevational gradient
Source: Aquat Sci. 2019 Apr 16;81(3):45. doi: 10.1007/s00027-019-0643-2 (PMC6469636; doi:10.1007/s00027-019-0643-2)
Supplement: Supplementary file 1 — Supplementary material 1 (DOCX 635 kb) [file 27_2019_643_MOESM1_ESM.docx]

# **Electronic Supplementary material**

# for

Importance of mixotrophic flagellates during the ice-free season in lakes located along an elevational gradient

Anna Waibel, Hannes Peter, and Ruben Sommaruga*

University of Innsbruck, Department of Ecology, Lake and Glacier Research Group, Technikerstr. 25, 6020 Innsbruck, Austria

*ruben.sommaruga@uibk.ac.at

Table S1. Geographical position and elevation of the sampled lakes.

Table S2. Results of the paired T-tests between data from July and October.

**Fig. S1.** Correlation matrix and trends for the environmental parameters along the elevational gradient. Data from both samplings (July and October) and both sample types (composite water sample and samples from the chl-a maximum) are combined. Panels below the diagonal show scatter plots and loess model fits for all pairwise combination of key environmental data. The upper panels provide pairwise regression statistics. Font size is related to the correlation coefficient for visual guidance.

**Fig. S2.** Chlorophyll- a and total dissolved nitrogen concentration in the composite and maximum chlorophyll-a samples along the elevational gradient.

**Fig. S3.** Abundance of heterotrophic (apochlorotic cells) and phototrophic (chlorophyll-a containing cells) flagellates along the elevational gradient in July and October. Shown are data for the composite water sample and for the depth of maximum chlorophyll a concentration. The lines represent locally estimated scatterplot smoothing (loess) fits to the data.

**Fig. S4.** Relative abundance of mixotrophic flagellates (mixotrophs as percentage of the abundance of phototrophic flagellates or phytoflagellates) in July and October for the depth of the maximum chlorophyll-a and the composite sample. The lines represent locally estimated scatterplot smoothing (loess) fits to the data.

Table S1. Geographical position and elevation of the sampled lakes.

| **Lake** | **Coordinates** | **Elevation [m a.s.l.]** |
| --- | --- | --- |
| Baggersee Rossau | 47°15’56’’ N, 11°26’47’’ E | 567 |
| Lansersee | 47°14’25’’ N, 11°25’0,6’’ E | 851 |
| Piburgersee | 47°11’42’’ N, 10°53’20’’ E | 913 |
| Wildsee Seefeld | 47°19’16’’ N, 11°11’30’’ E | 1180 |
| Brennersee | 47°1’5’’ N, 11°30’14’’ E | 1311 |
| Obernbergersee | 47°15’56’’ N, 11°26’47’’ E | 1590 |
| Seebensee | 47°22’6’’ N, 10°56′3″ E | 1657 |
| Drachensee | 47°21’36″ N, 10°56′0″ E | 1874 |
| Oberer Plenderlesee | 47°12’11″ N, 11°2′17″ E | 2344 |
| Gossenköllesee | 47°14’0″ N, 11°1′0″ E | 2417 |
| Mutterbergersee | 47°0'58" N, 11°7'41" E | 2483 |
| Schwarzsee ob Sölden | 46°57'53'' N, 10°56'48'' E | 2796 |

Table S2. Results of the paired T-tests between data from July and October.

| **Parameter** | **July vs. October** |  |
| --- | --- | --- |
|  | **t** | **p-value** |
| Temperature | 3.72 | 0.005 |
| PAR attenuation depth | -0.25 | 0.81 |
| DOC | -0.75 | 0.47 |
| TDP | 0.51 | 0.62 |
| DN | 1.21 | 0.25 |
| Chl-a | -1.15 | 0.29 |
| Bacterial abundance | 0.98 | 0.35 |
| Phytoplankton abundance | 0.36 | 0.72 |

**Supporting Figure S1**

**Supporting Figure S2**

**Supporting Figure S3**

**Supporting Figure S4**
